# The effect of corn silage hybrid and inclusion on performance of finishing steers and silage hybrid effects on digestibility and performance of growing steers

**DOI:** 10.1093/tas/txac147

**Published:** 2022-11-02

**Authors:** F Henry Hilscher, Curt J Bittner, Jana L Gramkow, Melissa L Jolly-Breithaupt, Mitch M Norman, Hannah C Wilson, Andrea K Watson, James C MacDonald, John N Anderson, Galen E Erickson

**Affiliations:** Department of Animal Science, University of Nebraska-Lincoln, Lincoln, NE 68583, USA; Department of Animal Science, University of Nebraska-Lincoln, Lincoln, NE 68583, USA; Department of Animal Science, University of Nebraska-Lincoln, Lincoln, NE 68583, USA; Department of Animal Science, University of Nebraska-Lincoln, Lincoln, NE 68583, USA; Department of Animal Science, University of Nebraska-Lincoln, Lincoln, NE 68583, USA; Department of Animal Science, University of Nebraska-Lincoln, Lincoln, NE 68583, USA; Department of Animal Science, University of Nebraska-Lincoln, Lincoln, NE 68583, USA; Department of Animal Science, University of Nebraska-Lincoln, Lincoln, NE 68583, USA; Dow AgroSciences, Mycogen Seeds, Indianapolis, IN 46268, USA; Department of Animal Science, University of Nebraska-Lincoln, Lincoln, NE 68583, USA

**Keywords:** brown midrib, corn silage, digestibility, feedlot, growing cattle

## Abstract

Three experiments evaluated the effects of three corn silage hybrids, inclusion, and nutrient digestibility in growing and finishing diets. The three hybrids tested included a control (CON), a hybrid containing a brown midrib (*bm3*) trait (BM3), and an experimental *bm3* hybrid with the soft endosperm trait (BM3-SOFT). Experiment 1 utilized 360 crossbred steers (body weight [BW] = 334; SD = 25 kg) to evaluate inclusion of silage in a finishing diet at (15% or 45% of diet dry matter [DM]) and silage hybrid (CON, BM3, or BM3-SOFT). Experiment 2 and 3 utilized 216 crossbred steers (BW = 324; SD = 10 kg) and six ruminally fistulated steers (BW = 274; SD = 27 kg), respectively, to evaluate effects of either CON, BM3, or BM3-SOFT silage hybrids on performance and nutrient digestibility in growing diets. In Exp. 1, there was a silage inclusion × hybrid interaction for average daily gain (ADG) and gain-to-feed ratio (G:F). All treatments with 15% silage had greater (*P* ≤ 0.04) ADG and G:F compared with 45% silage. Cattle fed BM3-SOFT had greater ADG and G:F than cattle fed CON or BM3 when silage was included at 15% of the diet. When silage was fed at 45% of the diet DM, ADG did not differ between cattle fed either *bm3* hybrid. Cattle fed BM3 had the greatest G:F (*P* < 0.01), with no difference between BM3-SOFT and CON. At 15% silage inclusion, hot carcass weight (HCW) was greater (*P* < 0.01) for cattle fed BM3-SOFT compared with cattle fed CON and BM3 but did not differ between cattle fed BM3 and CON. At 45% silage inclusion, steers fed either *bm3* hybrid did not differ in HCW but were both heavier (*P* < 0.01) compared with cattle fed CON. In Exp. 2, ending BW, dry matter intake (DMI), and ADG were greater (*P* < 0.01) for steers fed either *bm3* hybrid compared to steers fed the CON, but not different between steers fed the *bm3* hybrids. There were no differences (*P* = 0.26) in G:F between the silage hybrids. In Exp. 3, steers fed either *bm3* had greater (*P* < 0.01) neutral detergent fiber (NDF) and acid detergent fiber (ADF) digestibility than steers fed the CON. Ruminal pH was lower (*P* < 0.01), and total volatile fatty acid (VFA) concentration was greater (*P* < 0.01) for steers fed *bm3* hybrids compared to steers fed CON. Feeding silage with the *bm3* trait improved fiber digestibility, which increased DMI and subsequent ADG in high-forage growing diets. Feeding corn silage with the *bm3* trait improved performance compared to non-*bm3* corn silage when included above typical roughage concentration.

## INTRODUCTION

Changing the lignin content of the fiber portion of corn silage has the potential to change fiber digestibility of the plant in beef cattle diets (Tjardes et al., 2000). The lower lignin content of the brown midrib (*bm3*) mutation could be valuable to cattle fed high-forage diets as improving neutral detergent fiber (NDF) digestibility should increase dry matter intake (DMI) and average daily gain (ADG). The NDF of *bm3* silage has been shown to be more digestible in the rumen, have increased passage rate, and reduced rumen fill which could support greater DMI compared to conventional corn silage hybrids (Oba and Allen, 2000). When conventional corn silage hybrids replace corn in finishing diets, gain-to-feed ratio (G:F) decreases as corn silage increases in the diet (Goodrich et al., 1974; Burken et al., 2017a). While incorporating distillers grains and corn silage at higher inclusion levels in growing and finishing diets has shown to be more advantageous to corn silage alone (Felix et al., 2014; Burken et al., 2017a, 2017b), little research has been done in beef finishing and growing diets with corn silage incorporating the *bm3* trait and distillers grains. We hypothesize that feeding *bm3* silage may enhance animal performance by increasing fiber digestion and DMI in growing cattle and offset the negative effects of feeding greater inclusions of corn silage compared to traditional inclusions as a roughage in finishing cattle.

The objective of the following studies was to 1) evaluate two corn silage hybrids containing the *bm3* trait compared to a control silage fed at either 15% or 45% of diet DM with 20% distillers grains, 2) determine the effect of feeding two *bm3* corn silage hybrids on growing steer performance, and 3) determine digestibility and ruminal fermentation characteristics for two *bm3* corn silage hybrids in growing steers.

## MATERIALS AND METHODS

All animal use procedures were reviewed and approved by the University of Nebraska-Lincoln Institutional Animal Care and Use Committee.

### Corn Cultivation, Harvest, and Chemical Composition

Three hybrids of corn silage were grown in a single irrigated field at the Eastern Nebraska Research and Extension Center (ENREC) near Mead, NE. The three hybrids (Mycogen Seeds, Indianapolis, IN) were a standard corn silage hybrid which served as the control (CON; hybrid-TMF2R720), a *bm3* hybrid (BM3; hybrid-F15579S2), and an experimental *bm3* hybrid (BM3-SOFT; hybrid-F15578XT; Unified) with a softer endosperm trait SilaSoft (Mycogen Seeds). Planting density was targeted at 84,015 seeds per ha, and all seeds were grown under the same growing conditions. The field was managed as a corn, soybean, and wheat rotation for the previous 6 yr. Corn silage was harvested using a self-propelled forage harvester (JD 5400, John Deere, Moline, IL) set for a 1.27-cm theoretical length of chop, without a kernel processing unit.

Corn silage harvest initiation occurred on September 11, 2015 when the corn silage was at approximately ¾ milkline and whole plant corn silage samples were 35% dry matter (DM) determined by a moisture tester (Koster Crop Tester, Inc., Brunswick, OH) prior to harvest. Silage was harvested from September 11, 2015 through September 16, 2015. Silage was stored in concrete wall bunkers at the ENREC feedlot and covered first with a sheet of oxygen barrier film (SiloStop; Bruni Rimini Ltd, London, United Kingdom), and then a sheet of black and white plastic (Up North Plastics, Cottage Grove, MN) until the initiation of the trial. Bunker samples were tested for DM and fermentation analysis 28 d after harvesting to ensure proper ensiling by taking core samples of 1 m in depth every 15 m across the length of the bunker. Corn silage was sampled weekly (*n* = 27) during the feeding trial and dried in a forced-air oven at 60 °C for 48 h to determine DM (Table 1). Samples, composited by month (*n* = 7 wk) were analyzed by a commercial laboratory (DairyOne, Inc., Ithaca, NY) for fermentation analysis, starch, and water-soluble carbohydrates. Silage samples were also analyzed for crude protein (CP), NDF, acid detergent fiber (ADF), and lignin by monthly composites (*n* = 7) at a commercial laboratory (DairyOne, Inc.). Total metric tons of DM harvested per hectare were 19.9, 17.6, and 16.6 from CON, BMR, and BMR-SOFT, respectively. Corn silage yield was not able to be analyzed statistically because of the lack of replicated field plots.

**Table 1.** Nutrient and fermentation analysis of silage hybrids^1^ (DM basis)

Nutrient^2^	CON		BM3		BM3-SOFT	
	Mean	CV^3^	Mean	CV^3^	Mean	CV^3^
DM, %	33.3	6.2	33.2	5.4	34.1	5.7
CP, % of DM	8.6	3.4	9.6	7.8	9.1	3.9
NDF, % of DM	40.9	4.3	41.0	4.4	39.0	3.6
ADF, % of DM	27.1	2.5	26.7	2.2	23.6	3.0
Lignin, % of DM	4.3	27.5	3.7	24.2	2.81	34.6
Lignin, % of NDF	10.4	29.3	9.2	31.8	7.3	31.3
Starch, % of DM	31.0	8.8	32.0	8.9	30.8	6.7
Sugar, % of DM	2.3	28.1	2.4	37.8	2.8	22.4
pH	3.89	2.5	3.86	1.9	3.81	6.3
Lactic Acid, % of DM	5.6	17.1	6.2	16.6	6.0	15.6
Acetic acid, % of DM	1.4	31.2	1.6	30.9	1.5	34.4
Propionic acid, % of DM	0.34	40.5	0.43	48.7	0.46	54.0
Butyric acid, % of DM	<0.01	0.0	<0.01	0.0	<0.01	0.0
Total acids, % of DM	7.3	10.4	8.2	11.0	7.9	10.8

^1^Treatments were control (CON; hybrid-TMF2R720), a *bm3* hybrid (BM3; hybrid-F15579S2), and an experimental *bm3* hybrid (BM3-SOFT; hybrid-F15578XT) with a softer endosperm.

^2^DM was calculated using weekly samples (*n* = 27) and oven-dried for 48 h at 60 °C. All other samples are based on monthly composites (*n* = 7) of weekly samples taken during the finishing trial, and analyzed at Dairy One Labs (Ithaca, NY).

^3^CV = coefficient of variation and is calculated by dividing the standard deviation by the mean and is expressed as a percentage.

### Experiment 1: Cattle Finishing Experiment

Crossbred steers (*n* = 360; initial body weight [BW] = 334; SD = 25 kg) were purchased from auction markets as freshly weaned calves approximately 30 d prior to start of the experiment in multiple sources across a 10-d period. Prior to the initiation of the experiment, all steers were individually identified using both an electronic and two physical identification tags. Steers were processed upon arrival at the research feedlot and given a modified live viral vaccine for infectious bovine rhinotracheitis, bovine viral diarrhea types I and II, parainfluenza3, bovine respiratory syncytial virus, a *Mannheimia haemolytica–Pasteurella multocida* bacterin-toxoid (Titanium 5 PH-M, Elanco Animal Health, Greenfield, IN), and a *Haemophilus somnus* vaccine (Somnu Shield; Elanco Animal Health) administered at 2 mL per steer. They were treated for internal and external parasites with an injectable wormer (Dectomax; Zoetis Animal Health, Kalamazoo, MI) administered at 1 mL/45.4 kg of BW. All steers were revaccinated 28 d after initial processing with modified live viral vaccines for infectious bovine rhinotracheitis, bovine viral diarrhea types I and II, parainfluenza3, bovine respiratory syncytial virus (Titanium 5, Elanco Animal Health), and a killed viral vaccine for clostridial infections (Ultrabac 7, Zoetis Inc.).

Prior to the start of the experiment, all steers were limit-fed a common diet consisting of 50% alfalfa hay and 50% wet corn gluten feed (Sweet Bran, Cargill, Blair, NE) at 2.0% of projected BW for 5 d to equalize gastrointestinal fill (Watson et al., 2013) prior to weighing on day 0 and day 1 for initial BW determination (Stock et al., 1983). Steers were sorted into three BW blocks and assigned randomly to one of 36 pens (10 steers per pen) based on day 0 BW. The light block contained three replications, the middle BW block contained two replications, and the heaviest BW block contained one replication. Treatments were designed as a 2 × 3 factorial arrangement by varying the inclusion of corn silage in the finishing diet (15% or 45% silage on a DM basis) and silage hybrid (CON, BM3, or BM3-SOFT; Table 2). Corn silage fed at 45% of diet DM in the finishing diet replaced a 50:50 blend of dry-rolled and high-moisture corn compared to 15% silage treatments. Cattle were transitioned over a 24-d period. Dietary silage concentration of either 15% or 45% was held constant, while concentration of hay was decreased and 50:50 blend of dry-rolled and high-moisture corn was increased to final diet levels. Hay inclusions were the same for adaptation diets for both the 15% and 45% silage inclusions. All steers were fed a supplement formulated for 30 g per ton of monensin (Rumensin, Elanco Animal Health, DM basis) and a targeted intake of 90 mg per steer daily of tylosin (Tylan, Elanco Animal Health). Steers were implanted with Component TE-IS (80 mg trenbolone acetate and 16 mg estradiol; Elanco Animal Health) on day 1, and reimplanted with Component TE-200 (200 mg of trenbolone acetate and 20 mg estradiol; Elanco Animal Health) on day 91. Cattle were housed in open lots with 26 to 30 m^2^ of pen space per animal, and 30 to 39 cm of linear bunk space per steer. Steers had ad libitum access to fresh clean water and their respective diets. Steers were fed once daily at approximately 0730 h in concrete fence-line bunks with the same Roto-Mix model 420 (Roto-Mix, Doge City, KS) mixer/delivery box mounted on a single-axle feed truck for the duration of the study. Feed bunks were assessed at approximately 0530 h with the goal of trace amounts of feed at the time of feeding. Feed refusals were removed from feed bunks when needed, weighed, and subsampled. All feed refusals were subsampled and dried for 48 h in a 60 °C forced-air oven for determination of DM and calculation of refusal DM weight (AOAC, 1999, Method 935.29). Dietary ingredients were sampled weekly for determination of DM content. Dietary as-fed ingredient proportions were adjusted weekly. Cattle were fed from November 12, 2015 through May 10, 2016. All steers were fed for 181 d and were harvested at a commercial abattoir (Greater Omaha Packing, Omaha, NE) on May 11, 2016. On the day of shipping to the commercial abattoir, pens of steers were fed 50% of the previous day’s DM offering at regular feeding time. Pens of steers were then weighed on a platform scale at 1500 h prior to being loaded for shipping. A 4% pencil shrink was applied to this BW for final live BW and calculation of dressing percentage (hot carcass weight [HCW]/shrunk live final BW).

**Table 2.** Diet composition (% DM basis) in Exp. 1 to finishing cattle

	Treatments^1^					
	15% corn silage			45% corn silage		
Ingredient	CON	BM3	BM3-SOFT	CON	BM3	BM3-SOFT
Control corn silage	15.0	—	—	45.0	—	—
BM3 corn silage	—	15.0	—	—	45.0	—
BM3-SOFT corn silage	—	—	15.0	—	—	45.0
MDGS^2^	20.0	20.0	20.0	20.0	20.0	20.0
Dry-rolled corn	30.5	30.5	30.5	15.5	15.5	15.5
High-moisture corn	30.5	30.5	30.5	15.5	15.5	15.5
Supplement^3^						
Fine ground corn	1.3333	1.3333	1.3333	1.0833	1.0833	1.0833
Limestone	1.6750	1.6750	1.6750	1.6750	1.6750	1.6750
Salt	0.3000	0.3000	0.3000	0.0300	0.0300	0.0300
Urea	0.5000	0.5000	0.5000	0.7500	0.7500	0.7500
Tallow	0.1000	0.1000	0.1000	0.1000	0.1000	0.1000
Trace mineral premix^4^	0.0500	0.0500	0.0500	0.0500	0.0500	0.0500
Vitamin A-D-E premix^5^	0.0150	0.0150	0.0150	0.0150	0.0150	0.0150
Monensin^6^	0.0165	0.0165	0.0165	0.0165	0.0165	0.0165
Tylosin^7^	0.0102	0.0102	0.0102	0.0102	0.0102	0.0102

^1^Treatments were control (CON; hybrid-TMF2R720), a *bm3* hybrid (BM3; hybrid-F15579S2), and an experimental *bm3* hybrid (BM3-SOFT; hybrid-F15578XT) with a softer endosperm.

^2^MDGS = modified distillers grains with solubles.

^3^Supplement was formulated to be fed at 4.0% of diet DM.

^4^Trace mineral premix contained 6% Zn, 5.0% Fe, 4.0% Mn, 2.00% Cu, 0.29% Mg, 0.2% I, and 0.05% Co.

^5^Vitamin A-D-E premix contained 30,000 IU of vit A, 6,000 IU of vit D, 7.5 IU of vit E per gram.

^6^Monensin (Rumensin-90; Elanco Animal Health, Indianapolis, IN) premix contained 198 g/kg monensin.

^7^Tylosin (Tylan-40; Elanco Animal Health, Indianapolis, IN) premix contained 88 g/kg tylosin.

Hot carcass weight and liver scores were obtained the day of harvest. Liver abscesses were categorized from 0 (no abscesses), A−, A, or A+ (severely abscessed) according to the procedures outlined by Brink et al. (1990). Liver abscess categories were then combined to calculate the proportion of steers with or without abscessed livers in each pen. Carcass-adjusted final BW, used in calculation of ADG and G:F, was calculated from HCW and a 63.8% common dressing percentage. Marbling score, 12th rib fat thickness, and longissimus muscle (LM) area were recorded after a 48-h carcass chill. The energy value of the diets for net energy of maintenance (NEm) and net energy of gain (NEg) were calculated by utilizing pen data in the Galyean (2009) Net Energy calculator based on NRC (1996) net energy equations. The calculator utilizes initial BW, final BW, DMI, ADG, and target end point (assuming choice quality grade).

Performance and carcass data were analyzed using the MIXED procedure of SAS 9.2 (SAS Institute, Inc., Cary, NC) with pen serving as the experimental unit and block as a fixed effect. The treatment design was a 2 × 3 factorial; therefore, data were first evaluated for an interaction between hybrid and inclusion. If a significant interaction was observed for performance variables, then simple effects of hybrid within either 15% or 45% corn silage were evaluated. Significance of effects was determined at *P* ≤ 0.05.

### Experiment 2: Cattle Growing Experiment

A 76-d growing study was conducted from February 25, 2016 to May 10, 2016 utilizing 216 yearling crossbred steers (BW = 324; SD = 10 kg). Upon arrival and prior to initiation of the experiment, steers were identified and processed as previously described. Cattle were limit-fed a diet of 50% Sweet Bran and 50% alfalfa hay at 2.0% of projected BW for 5 d prior to trial initiation to equalize gut fill (Watson et al., 2013). Steers were weighed over two consecutive days, with the average of the first 2 d used as initial BW (Stock et al., 1983). Initial BW was calculated by averaging the 2-d weights. Cattle were stratified by BW and assigned randomly to pens with 12 head per pen. Pens were assigned randomly to one of three treatments, with six replications per treatment. The three corn silage hybrid treatments were set up in a generalized randomized design with 80% (diet DM) of the diet as either CON, BM3, or BM3-SOFT (Table 3). All diets included 15% modified distillers grains plus solubles (MDGS) and 5% supplement. Monesin (Elanco Animal Health) was added in the supplement to supply 200 mg per steer daily. Steers were treated for external parasites (StandGuard, Elanco Animal Health) and were implanted with Ralgro (36 mg zeranol, Merck Animal Health) on day 1. Feed bunks were assessed at approximately 0530 h and managed to allow for trace amounts of feed to remain at time of feeding. Cattle were housed in open lots with 26 to 30 m^2^ of pen space per animal, and 30 to 39 cm of linear bunk space per steer. Steers had ad libitum access to fresh clean water and their respective diets. Steers were fed once daily at approximately 0930 h in concrete fence-line bunks with the same Roto-Mix model 274 (Roto-Mix, Doge City, KS) mixer/delivery box mounted on a single-axle feed truck for the duration of the study. All feed refusals were removed from bunks when needed, weighed, and subsampled. All subsamples were dried for 48 h in a 60 °C forced-air oven for determination of DM and calculation of refusal DM weight. Dietary ingredients were sampled weekly for determination of DM content. Dietary as-fed ingredient proportions were adjusted weekly. Ending BW was collected similar to initial BW with steers limit-fed a diet of 50% Sweet Bran and 50% alfalfa hay at 2% of BW for 5 d and weighed for two consecutive days. The energy value of the diets was calculated by utilizing pen data in the Galyean (2009) Net Energy calculator based on NRC (1996) net energy equations. The calculator utilizes BW and composition of gain with a target end point at choice quality grade to calculate NEm and Neg of the diet based on DMI and ADG.

**Table 3.** Diet composition (% DM basis) in Exp. 2 fed to growing steers and Exp. 3 for nutrient digestion

Ingredient	Treatment^1^		
	CON	BM3	BM3-SOFT
Control corn silage	80.0	—	—
BM3 corn silage	—	80.0	—
BM3-SOFT corn silage	—	—	80.0
MDGS^2^	15.0	15.0	15.0
Supplement^3^			
Fine ground corn	3.0100	3.0100	3.0100
Limestone	0.9160	0.9160	0.9160
Salt	0.3000	0.3000	0.3000
Urea	0.5740	0.5740	0.5740
Tallow	0.1250	0.1250	0.1250
Trace mineral premix^4^	0.0500	0.0500	0.0500
Vitamin A-D-E premix^5^	0.0150	0.0150	0.0150
Monensin^6^	0.0100	0.0100	0.0100

^1^Treatments were control (CON; hybrid-TMF2R720), a *bm3* hybrid (BM3; hybrid-F15579S2), and an experimental *bm3* hybrid (BM3-SOFT; hybrid-F15578XT) with a softer endosperm.

^2^MDGS = modified distillers gains with solubles.

^3^Supplement was formulated to be fed at 4.0% of diet DM.

^4^Trace mineral premix contained 6% Zn, 5.0% Fe, 4.0% Mn, 2.00% Cu, 0.29% Mg, 0.2% I, and 0.05% Co.

^5^Vitamin A-D-E premix contained 30,000 IU of vit A, 6,000 IU of vit D, 7.5 IU of vit E per gram.

^6^Monensin (Rumensin-90; Elanco Animal Health, Indianapolis, IN) premix contained 198 g/kg monensin.

Performance data (BW, DMI, ADG, and G:F) were analyzed using the MIXED procedure of SAS 9.2 (SAS Institute, Inc.) with pen serving as the experimental unit. Significance was declared at *P* ≤ 0.05.

### Experiment 3: Steer Digestion Experiment

Six ruminally fistulated steers (BW = 290; SD = 27 kg) were used in a 3 × 6 Latin rectangle experiment to determine diet digestibility used in the steer growing experiment (Exp. 3). Steers were assigned randomly to the same three growing diets as described in Exp. 2 (CON, BM3, BM3-SOFT). Using six steers in a 3 × 6 design allowed for 12 observations per treatment. The study consisted of six periods that were 21 d in length with a 16-d adaptation period and a 5-d collection period. Steers were housed in individual slatted floor pens that were 3.70 m wide and 2.14 m in length. The study was conducted over 126 d.

Diets were mixed twice weekly and stored in a cooler held at 4 °C to ensure fresh feed was maintained. Steers were fed once daily at 0800 h. Feed refusals were removed daily prior to feeding. Refusals collected on day 17 to 21 were saved and dried in a forced-air oven at 60 °C for 48 h (AOAC, 1999, Method 935.29) to determine DM content. Individual feed ingredients were collected and dried in a 60 °C forced air oven weekly to ensure that accurate DM were used when mixing dietary treatments. Feeds offered and refused were analyzed for NDF (Van Soest and Marcus, 1964; Van Soest et al., 1991), ADF, acid detergent lignin (ADL; Van Soest, 1963), starch (AOAC, 2007, Method 996.11), and organic matter (OM; 600 °C for 6 h).

Titanium dioxide was ruminally dosed at 5 g per steer twice daily at 0700 and 1500 h on day 10 to 20. Fecal grab samples (approximately 300 g) were collected at 0700, 1100, 1500, and 1900 h during day 17 to 20 of each period. Fecal samples were composited on a wet basis into daily composites by steer, lyophilized (Virtis Freezemobile 25ES, Life Scientific, Inc., St. Louis, MO), and ground through a 1-mm screen using a Wiley mill (No. 4, Thomas Scientific, Swedesboro, NJ). The lyophilized and ground daily composites were then composited on a dry weight basis by steer within collection period. Fecal samples were subsequently analyzed for OM, NDF, ADF, ADL, and starch concentration using the procedures mentioned above. Titanium dioxide concentration of fecal samples was determined as described by Myers et al. (2004). Concentration of TiO_2_ was then used to calculate fecal DM output using the following equation (Cochran and Galyean, 1994): [(g marker dosed per day) ÷ (concentration of marker in feces)]. Total tract digestibility was calculated using the following equation (Cochran and Galyean, 1994): [(kg of nutrient fed − kg of nutrient refused − kg of nutrient in feces) ÷ (kg of nutrient fed − kg of nutrient refused)] × 100.

Rumen pH was recorded every minute using weighted wireless pH probes (Dascor, Inc., Escondido, CA) from day 17 to 20 of the collection period. Whole rumen contents were collected on day 21 of each period at 1400 h (6 h post-feeding). A sample of 250 mL of contents (in duplicate) was frozen for volatile fatty acids (VFAs; Trace 1300, Thermo Fisher Scientific Inc., Waltham, MA) using the procedures outlined by Ehrlich et al. (1981). Additional, rumen samples were incubated and stirred for 2, 4, and 6 h post-collection in a 39 °C incubated orbital shaker (Model 4730, Queue Systems, Parkersburg, WV) to determine VFA production. At the end of the designated time point, contents were removed from the incubator and frozen for determination of VFA. The difference of VFA concentration at 0, 2, 4, and 6 h was used to determine the rate of VFA production. At the time of analysis, rumen fluid samples were thawed in a cooler (4 °C) to ensure that no additional fermentation occurred. Each sample collected was analyzed twice for VFA concentration to ensure an accurate value was obtained. Additionally, from the whole rumen samples, a 150 g (as-is basis) sample of contents was placed into 250-mL glass bottles (in duplicate) and fitted with a gas production module (Ankom Technologies, Macedon, NY). The bottles were incubated in a 39 °C water bath where the modules recorded cumulative pressure (PSI) every 30 min for 20 h to determine rate of gas production. Gas production (mL) was determined using the ideal gas law (*n* = *p*[*V*/*RT*]) and Avogadro’s law (1 psi = 6.89 kilopascal) as described in the Ankom RF Gas Production System Operator’s Manual (Ankom Technologies, Macedon, NY).

Digestibility data were analyzed as a Latin rectangle using the MIXED procedure of SAS 9.2 (SAS Institute, Inc.) with period and treatment as fixed effects and steer as a random effect. Ruminal pH data were analyzed as repeated measures using the GLIMMIX procedure of SAS 9.2 with day as the repeated measure, treatment as a fixed effect, and steer as a random effect. Rumen VFA data were analyzed using the MIXED procedure of SAS 9.2, with fixed effects of period, treatment, hour, and interaction of hour by treatment, and steer as a random effect. Gas production data were analyzed using the MIXED procedure of SAS 9.2. Response variables were total gas production and gas production rate. Bottle served as the experimental unit. Rate of gas production was generated by analyzing the gas production data in a modified Gompertz model (Schofield et al., 1994; Huhtanen et al., 2008) using the NLIN procedure of SAS 9.2. Significance of effects was determined at (*P* ≤ 0.10).

## RESULTS AND DISCUSSION

### Corn Silage

Corn silage was targeted to be harvested at 35% DM (Table 1). The fermentation analysis of the three corn silage hybrids indicated that proper fermentation did occur as pH was below 3.9, as well as having total acids greater than 7.3% (Cherney and Cherney, 2003). The starch percentage and the sugar (water-soluble carbohydrates) percentage remained consistent across all three silage hybrids. The ADF and lignin concentrations were numerically lower in both the BM3 and BM3-SOFT compared to the CON, as expected.

### Experiment 1: Cattle Finishing Experiment

There was a silage inclusion by hybrid interaction (*P* ≤ 0.05); therefore, simple effects will be presented (Table 4). No interaction was observed between hybrid and inclusion for DMI. Cattle fed 45% silage averaged across hybrids had greater DMI (*P* < 0.01) compared to steers fed 15% silage. Corn silage hybrid did not significantly affect (*P* = 0.11) DMI. Keith et al. (1981) reported that as silage inclusion increased in finishing diets, DMI increased. The authors also reported that at greater inclusions of corn silage, cattle fed *bm3* corn silage had greater DMI than cattle fed non-*bm3* corn silage, but at low inclusion there was no difference in DMI between *bm3* and non-*bm3* corn silage. DiCostanzo et al. (1997) fed finishing diets containing 12%, 24%, 36%, or 48% corn silage; these researchers reported that there was a linear increase in DMI as corn silage inclusion increased in the diet. Burken et al. (2017b) conducted two feeding trials comparing the silage inclusion of 15% or 45% in finishing cattle. In the first experiment, there were no differences in DMI across treatments, while in the second trial, increasing the silage from 15% to 45% increased DMI 12.0 vs. 12.2 kg/d for the 15% and 45% silage levels, respectively. In this experiment, cattle fed BM3-SOFT had greater ADG than CON or BM3 when silage was included at 15% of the diet. When silage was fed at 45% of the diet DM, cattle fed BM3 and BM3-SOFT did not differ in ADG, but both were greater than CON (*P* < 0.05). Interestingly, steers fed BM3 and BM3-SOFT at 45% of the diet did not differ in ADG to steers fed either 15% CON or 15% BM3 suggesting the *bm3* trait allowed for more silage to be fed without compromising ADG. All treatments with 15% corn silage inclusion had greater (*P* ≤ 0.04) G:F compared to 45% corn silage inclusion, but G:F response due to hybrid was different depending on inclusion. For steers fed 15% silage, G:F was greatest for BM3-SOFT, lowest for BM3, and intermediate for CON. The range in G:F across the hybrids was 0.174 for BM3-SOFT to 0.166 for BM3. For steers fed 45% silage, G:F was greatest for cattle fed BM3 while CON and BM3-SOFT were not different. The range in G:F was 0.162 for BM3 to 0.154 for BM3-SOFT. Similar to G:F, dietary net energy for maintenance (NEm) and net energy for gain (NEg) values were greater (*P* ≤ 0.01) for cattle fed 15% corn silage compared to 45% corn silage inclusion, but differed between hybrid depending on inclusion. At 15% corn silage, NEm and NEg were greatest for the BM3-SOFT and lowest for the BM3 hybrid, while at 45% corn silage BM3 had the greatest NEm and NEg with CON being the lowest. Keith et al. (1981) compared the performance of feedlot cattle fed either *bm3* or non-*bm3* silage at inclusions of 88%, 60%, and 27% on DM basis in finishing diets. Cattle fed *bm3* at both 88% and 60% of diet DM had greater total gain and ADG compared to the non-*bm3*-fed cattle. Cattle fed *bm3* at the greater inclusion also had a tendency for an improvement in G:F compared to non-*bm3*-fed cattle. As inclusion of corn silage decreased in the finishing diet to 27%, no differences in feedlot performance were reported between the *bm3*- and non-*bm3*-fed cattle. McEwen and Buchanan-Smith (1996) compared a *bm3* hybrid with other commercial hybrids and these authors reported that cattle fed *bm3* silage did not differ in ADG and had greater G:F compared to other commercial hybrids.

**Table 4.** The effects of silage inclusion and silage hybrid on feedlot performance and carcass characteristics in finishing steers fed 181 d (Exp. 1)

Item	Treatments^1^									
	15% corn silage			45% corn silage			SEM	Int.^2^	Incl.^3^	Hybrid^4^
	CON	BM3	BM3-SOFT	CON	BM3	BM3-SOFT				
Pens, *n*	6	6	6	6	6	6				
Feedlot performance										
Initial BW, kg	334	333	334	333	334	334	0.3	0.49	0.57	0.36
Final BW^5^, kg	627^b^	626^b^	638^a^	608^c^	623^b^	623^b^	3.0	0.04	<0.01	<0.01
Live final BW, kg	625	623	630	618	622	623	2.9	0.49	0.03	0.15
DMI, kg/d	9.8	10.0	9.9	10.1	10.2	10.4	0.1	0.19	<0.01	0.11
ADG^5^, kg	1.66^b^	1.66^b^	1.73^a^	1.56^c^	1.64^b^	1.64^b^	0.02	0.05	<0.01	<0.01
G:F^5^	0.170^a^	0.166^b^	0.174^a^	0.154^d^	0.162^c^	0.157^d^	0.002	0.01	<0.01	0.07
NEm, Mcal/kg DM^6^	2.03^a,b^	2.00^b^	2.07^a^	1.86^d^	1.93^c^	1.88^c,d^	0.02	0.01	<0.01	0.16
NEg, Mcal/kg DM^6^	1.37^a,b^	1.35^b^	1.41^a^	1.22^d^	1.29^c^	1.24^c,d^	0.02	0.01	<0.01	0.13
Carcass characteristics										
HCW, kg	400^b^	399^b^	407^a^	388^c^	397^b^	398^b^	2.0	0.04	<0.01	<0.01
Dress, %	64.05^b^	64.15^a,b^	64.64^a^	62.75^c^	63.89^b^	63.87^b^	0.19	0.03	<0.01	<0.01
LM area, cm^2^	87.1	87.7	87.7	89.3	90.3	87.1	0.7	0.08	0.11	0.29
12th rib fat, cm	1.42	1.40	1.50	1.19	1.24	1.32	0.05	0.76	<0.01	0.23
Marbling score^7^	451	455	475	413	425	443	10.0	0.90	<0.01	0.03

^1^Treatments were control (CON; hybrid-TMF2R720), a *bm3* hybrid (BM3; hybrid-F15579S2), and an experimental *bm3* hybrid (BM3-SOFT; hybrid-F15578XT) with a softer endosperm.

^2^Silage inclusion × silage hybrid interaction.

^3^Fixed effect of silage inclusion.

^4^Fixed effect of silage hybrid.

^5^Calculated from HCW, adjusted to a common dressing percent of 63.8%.

^6^NEm and NEg were calculated using methodology of NRC (1996) using a tool developed by Galyean (2009), assuming a 624 kg target end point.

^7^Marbling score 400 = small^00^, 500 = modest^00^.

^a–d^Means with different superscripts differ (*P* < 0.05).

At 15% inclusion, BM3-SOFT had greater ADG and G:F compared to BM3; however, at 45% inclusion both treatments did not differ in ADG while BM3-SOFT had greater G:F. In comparing kernel type, Jaeger et al. (2006) reported that corn with softer endosperm had greater G:F compared to corn with harder endosperm when fed as dry-rolled corn (DRC) to finishing cattle; however, kernel moisture can impact performance. Macken et al. (2003) compared floury and flinty hybrids in finishing diets as DRC or high-moisture corn (HMC). When fed as DRC, corn with floury endosperm had greater G:F, but when fed as HMC there were no differences in G:F between floury and flinty hybrids. Szasz et al. (2007) reported no differences in ruminal or total tract starch digestibility between floury and flinty corn when fed as HMC. Utilizing silage hybrids similar to the current experiment, Grant et al. (2017) compared an isogenic control to a *bm3* hybrid and a *bm3* hybrid with a softer endosperm (*bm3*-*E*) fed to dairy cows fed 49% of the diet. These authors reported that the *bm3* and *bm3-E* had greater milk yield and fat corrected milk yield when compared to the control. Efficiency of milk production was greatest for *bm3-E* with the control having the lowest and the *bm3* being intermediate (Grant et al., 2017). Burken et al. (2017a) reported a linear decrease in NEm and NEg as corn silage inclusion increased in finishing diets.

At 15% corn silage inclusion, carcass-adjusted final BW and HCW were greater (*P* < 0.01) for BM3-SOFT compared to CON and BM3, but did not differ between BM3 and CON. At 45% corn silage inclusion, steers fed BM3-SOFT and BM3 did not differ in carcass-adjusted final BW and HCW but were both heavier (*P* < 0.01) compared to CON. Steers fed 15% silage had heavier (*P* < 0.01) carcass-adjusted final BW and HCW compared to steers fed 45% inclusion across hybrids. No significant interaction was observed for final live BW (*P* = 0.49). When CON silage was fed at 45% of diet DM, live final BW was reduced 7 kg compared to feeding CON at 15% inclusion. However, HCW was reduced by 12 kg when CON silage was fed at 45% compared to 15%. This relative change in HCW compared to final live BW illustrates the negative effect of increasing silage inclusion from 15% to 45% of diet DM on dressing percentage and gut fill. Dressing percentage at 15% inclusion was greatest (*P <* 0.03) for BM3-SOFT and lowest for CON with BM3 being intermediate. However, at 45% silage inclusion, steers fed both BM3-SOFT and BM3 had dramatically greater (*P* < 0.01) dressing percentages than CON suggesting less gut fill. All cattle fed 15% silage had greater (*P* < 0.01) dressing percentages compared to cattle fed 45% corn silage. When cattle are fed elevated inclusions of corn silage, dressing percentage decreases due to increased gut fill.

Burken et al. (2017a) reported a linear decrease in final BW and HCW as corn silage was increased in finishing diets. Additional studies by Burken et al. (2017b) reported a tendency for decreased final BW and HCW in Exp. 1 and a significant decrease in final BW and HCW in Exp. 2 as inclusion of corn silage increased from 15% to 45% of the diet. Peterson et al. (1973) reported that as corn silage inclusion increased, dressing percentage linearly decreased. Similarly, Brennan et al. (1987) reported cattle fed increased inclusions of corn silage had decreased dressing percentages. Cattle fed 15% corn silage had greater (*P* < 0.01) fat thickness over the 12th rib and marbling score compared to steers fed 45% corn silage in the finishing diet. Burken et al. (2017a) reported a linear decrease in dressing percentage and 12th rib fat thickness as corn silage inclusion increased. Tjardes et al. (2000) reported no differences in final BW, HCW, or any other carcass characteristics between cattle fed *bm3* compared to non-*bm3* corn silage over a 112-d growing period followed by finishing on a common diet. Keith et al. (1981) also reported no differences in yield and quality grade between cattle fed *bm3* and non-*bm3* corn silage when fed at high and low inclusions of silage. Replacing corn grain with corn silage in finishing diets resulted in decreased animal performance as energy content of the diet decreased (Keith et al., 1981). Intake increased as a result of this decrease in dietary energy to increase total energy intake. With the incorporation of *bm3* hybrids that are lower in lignin content, increased ruminal NDF digestion and passage rate allows for greater DMI, which could allow for greater energy intake, that minimizes the decrease in ADG, and G:F as silage inclusion increased from 15% to 45% compared to control silage.

### Experiment 2: Cattle Growing Experiment

Ending BW was greater (*P* < 0.01) for steers fed the BM3 and BM3-SOFT compared to the CON, but not different between the two *bm3* varieties (Table 5). Steers fed both BM3 and BM3-SOFT had greater (*P* < 0.01) DMI and ADG compared to the steers on the CON treatment, but DMI and ADG were not different between steers on the BM3 or BM3-SOFT treatments. While BM3 and BM3-SOFT had greater DMI and ADG, there were no differences (*P* = 0.26) in G:F among the three silage treatments. Calculated NEm and NEg values were not different (*P* ≥ 0.82) across all three treatments. Weller and Phipps (1986) compared *bm3* corn silage to non-*bm3* control fed to weaned heifer calves for 56 d. The authors reported that DMI was not different between *bm3* and non-*bm3*, but the calves fed *bm3* had 11% greater ADG which translated into improved G:F. Tjardes et al. (2000) evaluated a *bm3* hybrid to isogenic control in a 112-d growing trial with steers. In that study, during the growing phase, silage was fed at 86% of the diet DM. The authors reported that during the growing phase, DMI was greater for steers fed *bm3* than non-*bm3*, but there were no differences in ADG between the two treatments. Subsequently, G:F was greater for steers fed *bm3* during the growing phase. Tjardes et al. (2000) reported no differences in NEm and NEg values in silage growing diets between *bm3* and non-*bm3* hybrids. Saunders et al. (2015) compared a *bm3* hybrid to an isogenic control silage using individually fed cross bred beef steers. The authors reported that final BW tended to be greater at the end of the 84-d growing period. Steers fed *bm3* silage had a tendency for greater ADG and G:F compared to non-*bm3* silage with no difference in DMI between silage treatments (Saunders et al., 2015). Keith et al. (1981) compared the performance of cattle fed either *bm3* or non-*bm3* silage at inclusions of 88%. The authors reported that cattle fed *bm3* had greater total gain, DMI, and ADG compared to the non-*bm3*-fed cattle. Cattle fed *bm3* also had a tendency for an improvement in G:F compared to non-*bm3*-fed cattle (Keith et al., 1981). With high inclusions of forage in growing diets, bulk fill can limit intake and energy intake. The decreased lignin content of *bm3* hybrids allows for a greater percentage of digestible NDF, which in turn allows for increased passage rate allowing for greater intake. This increase in DMI allows for greater energy intake and translates to improved ADG, which agrees with the current study and previous research.

**Table 5.** Effects of feeding two different *bm3* corn silage hybrids on growing steer performance fed 76 d (Exp. 2)

Variable	Treatments			SEM	*P*-value
	CON	BM3	BM3-SOFT		
Pens, *n*	6	6	6		
Initial BW, kg	324	324	324	0.3	0.80
Ending BW, kg	449^b^	469^a^	468^a^	2.2	<0.01
DMI, kg/d	9.6^b^	10.9^a^	10.9^a^	0.1	<0.01
ADG, kg	1.64^b^	1.92^a^	1.90^a^	0.03	<0.01
G:F	0.171	0.176	0.174	0.002	0.26
NEm, Mcal/kg DM^2^	1.78	1.79	1.77	0.02	0.82
NEg, Mcal/kg DM^2^	1.15	1.16	1.15	0.02	0.90

^1^Treatments were control (CON; hybrid-TMF2R720), a *bm3* hybrid (BM3; hybrid-F15579S2), and an experimental *bm3* hybrid (BM3-SOFT; hybrid-F15578XT) with a softer endosperm.

^2^NEm and NEg were calculated using methodology of NRC (1996) using a tool developed by Galyean (2009) assuming a 635 kg target end point.

^a,b^Means with different superscripts differ (*P* < 0.05).

### Experiment 3: Steer Digestion Experiment

Feeding corn silage with the *bm3* trait tended to increase (*P* = 0.11) DMI and OM intake compared to CON (Table 6), this was also observed in Exp. 2 with identical diets fed to growing steers. Digestibility of DM tended to be impacted by treatment (*P* = 0.11) with steers fed BM3 and BM3-SOFT having greater DM digestibility than steers fed CON. Digestibility of OM was impacted by treatment (*P* = 0.05), with steers fed BM3-SOFT having greater OM digestibility than steers fed CON and steers fed BM3 being intermediate. There were significant differences in NDF excretion and NDF digestibility due to treatment (*P* < 0.01). Steers fed both BM3 (57.8%) and BM3-SOFT (57.0%) had greater (*P* < 0.01) NDF digestibility compared to the CON (45.3%). Intake of ADF was greatest (*P* = 0.03) for BM3 and lowest for BM3-SOFT with CON being intermediate. However, there were no differences (*P >* 0.10) in ADF digestibility between BM3 (59.6%) and BM3-SOFT (56.1%), but both had greater (*P* < 0.01) ADF digestibility than CON (41.9%). Cattle fed the BM3 treatment excreted the greatest (*P* = 0.03) amount of starch and CON excreted the least amount of starch. Starch digestibility was greater than 94.5% for cattle fed all three silages, but steers fed CON (96.6%) corn silage had the greatest (*P* = 0.03) starch digestibility with BM3-SOFT (95.8%) being intermediate and BM3 (94.6%) having the least starch digestibility. In a meta-analysis, Ferraretto and Shaver (2015) compared different hybrid types on lactation performance and total tract digestibility in dairy cows. These authors reported that *bm3* hybrids had greater DMI than dual purpose and leafy hybrids and DMI did not differ compared to high fiber digestibility hybrids that did not have the brown midrib trait. Ferraretto and Shaver (2015) reported no differences in DM or OM total tract digestibility between all four hybrids evaluated; however, the *bm3* and the high fiber digestibility hybrids had the greatest total tract NDF digestibility and the lowest total tract starch digestibility when compared to dual purpose and leafy hybrids. Intake can impact passage rate and in turn, passage rate can affect total tract digestibility. Oba and Allen (2000) reported that cows fed *bm3* hybrids had greater DMI when fed at low and high levels compared to an isogenic control, but there were no differences in total tract NDF digestibility. The authors did measure rumen passage and digestion rates, and while total tract NDF digestibility was not different, NDF passage rate for *bm3*-fed cattle was faster by about 8% compared to controls. In agreement with the current study, Weller and Phipps (1986) utilized sheep feed at maintenance and reported that sheep fed a *bm3* vs. a conventional silage hybrid had greater DM, OM, NDF, and ADF digestibility. Muller et al. (1972) compared just the stover fraction (ears removed prior to ensiling) of *bm3* and non-*bm3* hybrids in sheep. Lambs fed *bm3* silage had greater DMI, DM, NDF, and ADF digestibility compared to lambs than the controls. Tjardes et al. (2000) reported greater DMI and increases of 10.5 and 9.4 percentage unit improvements in total tract digestibility of NDF and ADF, respectively, for steers fed *bm3* hybrid compared to a control, but no differences in starch digestibility. Endosperm type had no effect on NDF digestibility. In HMC, vitreousness of grain did not affect animal performance when compared to DRC (Szasz et al., 2007). With the addition of moisture and fermentation, the proteins are solubilized and starch digestibility increases in HMC with greater moisture content (Owens, 2008). As corn grain in corn silage is harvested wetter than HMC, endosperm type may not impact corn silage starch digestibility. Grant et al. (2017) compared an isogenic control to a *bm3* hybrid and a *bm3* hybrid with a softer endosperm (*bm3*-*E*) fed to dairy cows. The authors reported that DMI was greatest for the *bm3* hybrid and lowest for the control with the *bm3*-*E* being intermediate. However, total tract digestibility was not different for OM, NDF, and starch among all three treatments (Grant et al., 2017). The general improvements in NDF, ADF, and OM digestibility for steers fed BM3 and BM3-SOFT likely explain the greater DMI observed in Exp. 3, as well as the greater gain observed in Exp. 2.

**Table 6.** Effects of feeding two different *bm3* corn silage hybrids on intake and digestibility of nutrients (Exp. 3)

Item	Treatments^1^				*P*-value
	Control	BM3	BM3-SOFT	SEM	
DM					
Intake, kg/d	6.8	7.5	7.4	0.5	0.11
Excreted, kg/d	2.40	2.45	2.22	0.18	0.39
Digestibility, %	64.5	67.7	69.0	1.6	0.11
OM					
Intake, kg/d	6.3	6.9	6.9	0.45	0.11
Excreted, kg/d	2.09	2.09	1.91	0.14	0.36
Digestibility, %	66.8^b^	70.0^a,b^	71.6^a^	1.4	0.05
NDF					
Intake, kg/d	2.67	2.94	2.75	0.18	0.08
Excreted, kg/d	1.45^b^	1.23^a^	1.17^a^	0.09	0.01
Digestibility, %	45.3^b^	57.8^a^	57.0^a^	2.2	<0.01
ADF					
Intake, kg/d	1.68^a,b^	1.81^a^	1.59^b^	0.09	0.03
Excreted, kg/d	0.95^b^	0.73^a^	0.68^a^	0.05	<0.01
Digestibility, %	41.9^b^	59.6^a^	56.1^a^	2.5	<0.01
Starch					
Intake, kg/d	2.03	2.09	2.29	0.18	0.11
Excreted, kg/d	0.07^b^	0.11^a^	0.09^a,b^	0.01	0.03
Digestibility, %	96.6^a^	94.6^b^	95.8^a,b^	0.7	0.03

^1^Treatments were control (CON; hybrid-TMF2R720), a *bm3* hybrid (BM3; hybrid-F15579S2), and an experimental *bm3* hybrid (BM3-SOFT; hybrid-F15578XT) with a softer endosperm.

^a,b^Means with different superscripts differ (*P* < 0.05).

There was a significant decrease (*P* < 0.01) in average ruminal pH between the *bm3* hybrids (6.24) and the control silage (6.50; Table 7). Additionally, the BM3 and BM3-SOFT treatments had lower (*P* < 0.01) maximum pH and lower (*P* < 0.01) minimum pH compared to the CON. The molar proportions of acetate were greatest (*P* < 0.01) in CON lowest for the BM3 treatment with BM3-SOFT being intermediate. The CON (22.38) and BM3-SOFT (22.60) treatments had lower (*P* < 0.01) molar proportions of propionate compared to the BM3 (23.73). The BM3 and BM3-SOFT cattle did have greater (*P <* 0.01) proportions of butyrate compared to CON. The BM3 treatment had a lower (*P* = 0.02) acetate to propionate ratio (2.70) compared to BM3-SOFT (2.85). The increase in propionate and lower acetate to propionate ratio for BM3 compared to BM3-SOFT suggests improved starch digestion with less energy losses for this relatively small shift in VFA profile. Lower pH and changes in VFA molar proportions for *bm3* silage may be related to greater fermentation and improved rumen digestibility and is further supported by greater (*P* < 0.01) total VFA concentrations compared to the control silage. The production rate of total VFA from whole rumen contents when collected at peak fermentation showed numerical increases in VFA production rate over 6 h for the BM3 and BM3-SOFT compared to CON, and were numerically greatest for the BM3 treatment (Table 8). While rate of acetate production was not different (*P* = 0.40) among hybrids, propionate production was greatest (*P* ≤ 0.03) for BM3 compared to CON and BM3-SOFT. Butyrate production was greatest for BM3 and BM3-SOFT compared to CON but not different between *bm3* hybrids. Gas production rates of whole rumen contents when collected at peak fermentation showed a significant increase over 20 h for the BM3 and BM3-SOFT compared to CON (*P* = 0.03) but were not different between *bm3* varieties. In agreement with the current study, Oba and Allen (2000) and Saunders et al. (2015) reported average pH was significantly lower for *bm3* hybrids compared to controls. Hassanat et al. (2017) reported that minimum pH was lower for *bm3* compared to a conventional corn silage which agrees with the current study. However, these authors reported no differences in average and maximum pH were observed between the *bm3* and non*-bm3* which differs from the current study. In contrast to these results, Tjardes et al (2000) reported that pH from *bm3*-fed steers was not significantly different from steers fed isogenic controls. In agreement with the current study, Weller and Phipps (1986) reported that feeding *bm3* silage resulted in a lower concentration of acetate and a greater concentration of propionate, which resulted in a decreased acetate to propionate ratio when compared to a non-*bm3* hybrid. Saunders et al. (2015) reported greater concentrations of total VFA, and propionate while a decrease in acetate concentration resulting in a lower acetate to propionate ratio when steers were *bm3* hybrids to a conventional corn silage control. While Tjardes et al. (2000) reported greater concentrations of total VFA, the authors also reported greater concentrations of acetate, and no differences in propionate concentrations between *bm3* and isogenic control hybrids. Lopes et al. (2009) reported that corn containing floury endosperm had lower rumen pH and acetate concentrations while propionate concentration was increased when fed as DRC. However, when harvested as corn silage and fermented, Fanning (2002) reported no differences in molar concentration of acetate, propionate, or total VFA concentration between floury and flinty hybrids. The BM3-SOFT with softer endosperm improved starch digestibility compared to BM3 but there was no difference between BM3 and BM3-SOFT for OM, NDF, or ADF digestibility. However, feeding corn silage hybrids with the *bm3* trait at 80% of the diet DM resulted in greater fiber and OM digestion compared to corn silage without the trait. Based on rumen pH, VFA concentration, and VFA and gas production data, greater fermentation occurred for cattle fed corn silage with the *bm3* trait compared to a control corn silage without the *bm3* trait.

**Table 7.** Effects of feeding two different *bm3* corn silage hybrids on rumen pH measurements and ruminal VFA concentration (Exp. 3)

Item	Treatments^1^			SEM	*P*-value
	CON	BM3	BM3-SOFT		
Ruminal pH					
Maximum pH	6.64^b^	6.37^a^	6.41^a^	0.07	<0.01
Average pH	6.50^b^	6.22^a^	6.26^a^	0.07	<0.01
Minimum pH	6.38^b^	6.08^a^	6.12^a^	0.07	<0.01
Magnitude	0.26^b^	0.29^a^	0.29^a^	0.17	<0.01
Variance	0.60^b^	0.85^a^	0.90^a^	0.11	<0.01
Ruminal VFA^2^					
Total VFA (mM)	182.0^b^	200.2^a^	193.6^a^	5.75	<0.01
Acetate^3^	62.1^a^	59.6^c^	61.1^b^	0.67	<0.01
Propionate^3^	22.4^b^	23.7^a^	22.6^b^	0.67	0.01
Butyrate^3^	10.7^b^	12.3^a^	12.3^a^	0.27	<0.01
A:P ratio^4^	2.83^a,b^	2.70^b^	2.85^a^	0.10	0.06

^1^Treatments were control (CON; hybrid-TMF2R720), a *bm3* hybrid (BM3; hybrid-F15579S2), and an experimental *bm3* hybrid (BM3-SOFT; hybrid-F15578XT) with a softer endosperm.

^2^Ruminal VFAs.

^3^VFA concentration in mol/100 mol.

^4^Acetate:propionate.

^a–c^Means with different superscripts differ (*P* < 0.05).

**Table 8.** Effects of feeding two different *bm3* corn silage hybrids on ruminal VFA and gas production rates (Exp. 3)

Production rate, mM/g DM^2^	Treatments^1^			SEM	*P*-value
	CON	BM3	BM3-SOFT		
Total VFA	41.79	55.10	49.14	5.04	0.17
Acetate	26.32	31.81	27.23	3.15	0.40
Propionate	7.25^b^	12.15^a^	9.00^b^	1.71	0.02
Butyrate	6.18^b^	10.13^a^	9.24^a^	1.21	0.03
Gas production rate, %/h^3^	25.43^b^	30.87^a^	28.73^a,b^	2.44	0.03

^1^Treatments were control (CON; hybrid-TMF2R720), a *bm3* hybrid (BM3; hybrid-F15579S2), and an experimental *bm3* hybrid (BM3-SOFT; hybrid-F15578XT) with a softer endosperm.

^2^Production rates is calculated by change in VFA mM/g DM over 6 h.

^3^Gas production rates.

^a,b^Means with different superscripts differ (*P* < 0.05).

Feeding corn silage with the *bm3* trait improved performance compared to non-*bm3* corn silage when fed at 45% by offsetting the negative effects of feeding greater inclusions of corn silage by reducing gut fill and increasing DMI but was variable between the *bm3* traits when fed at 15% inclusion. Feeding silage with the *bm3* trait in growing diets improved the rumen environment allowing for enhanced fiber digestion, which increased DMI and subsequent ADG.
